# Incidence of new-onset cardiac arrhythmias in cocaine and cannabis users: a retrospective cohort study

**DOI:** 10.1093/ehjacc/zuag011

**Published:** 2026-02-05

**Authors:** Laith Rhabneh, Ra’ed Ababneh, Shahd Qaddour, Abdel Rahman Alwardat, Mohammed Aloqaily, Khalid Hazaimeh, Ali Awad

**Affiliations:** Department of Internal Medicine, Hackensack Meridian Ocean University Medical Center, Brick Township, NJ 08724, USA; Department of Internal Medicine, Hackensack Meridian Ocean University Medical Center, Brick Township, NJ 08724, USA; Diagnostic Radiology Department, King Abdullah University Hospital, Ramtha, Irbid, Jordan; Department of Internal Medicine, King Abdullah University Hospital, Ramtha, Irbid, Jordan; Internal Medicine Department, University of Maryland Midtown Campus, Baltimore, MD, USA; Internal Medicine Department, Royal Jordanian Medical Services, Amman, Jordan; Detroit Medical Center, Wayne State University, Detroit, MI, USA

**Keywords:** Cocaine use disorder, Cannabis use disorder, Cardiac arrhythmia

## Abstract

**Aims:**

Cocaine use disorder prevalence is around 2.2% for individuals age 12 and older, with higher rates reaching 6.2% in young adults ages 18–25. In 2021, around 23% of all US overdose deaths were related to cocaine. Cannabis use disorder is more prevalent than cocaine use disorder with prevalence reaching 14.7%. This study aims to evaluate the impact of cocaine and cannabis on clinical cardiovascular outcomes. Specifically, it investigates the incidence of new-onset cardiac arrhythmia between the cocaine and cannabis users, as well as the incidence of cardiac arrest events, major adverse cardiovascular events including myocardial infarction (MI) and stroke, and all-cause mortality.

**Methods and results:**

We did a retrospective cohort analysis using the TriNetX database of cocaine and cannabis user patients and created two cohorts: cocaine and cannabis. Propensity score matching was employed to reduce baseline disparities. The primary outcome was the incidence of new-onset cardiac arrhythmia. Secondary outcomes included cardiac arrest events, all-cause mortality, and occurrence of major adverse cardiovascular events (MI and stroke). After matching, 248 769 patients were included in each cohort (cocaine and cannabis users). New-onset cardiac arrhythmia occurred more frequently in the cocaine group (0.2%) compared with the cannabis group (0.15%) (HR: 1.067; 95% CI: 1.022–1.115, *P* < 0.035). Cocaine use was also associated with higher rates of the secondary outcomes: cardiac arrest (HR: 1.442; 95% CI: 1.346–1.545, *P* < 0.001), all-cause mortality (HR: 1.215; 95% CI: 1.187–1.243, *P* < 0.013), and major adverse cardiovascular events (HR: 1.147; 95% CI: 1.116–1.178, *P* < 0.001).

**Conclusion:**

Cocaine users experience significantly higher rates of new-onset cardiac arrhythmias, cardiac arrest, and major adverse cardiovascular events (MI and stroke) compared with cannabis users. These findings highlight the differing outcomes associated with substance use. Future research should aim to validate our findings through prospective, multicentre studies with standardized diagnostic methods as well as longer-term follow-up to more accurately define the outcomes.

## Graphical Abstract

**Graphical Abstract:**
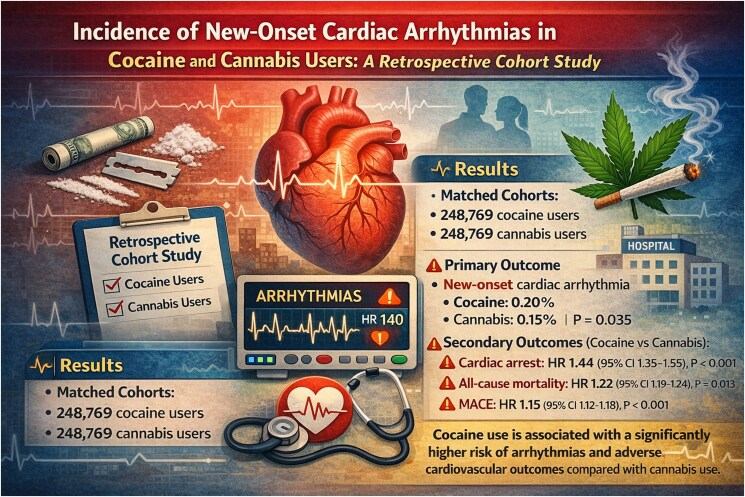

## Introduction

Substance use disorder is a common and growing public health problem in the USA.^1^ The prevalence of cocaine use disorder is 2.2% for those 12+ years old, with higher rates reaching to 6.2% in young adults ages 18–25.^2^ In 2021, around 23% of all US overdose deaths were related to cocaine.^3^ Cannabis use disorder is even more common, affecting an estimated 14.7% of the population, reflecting increasing legalization and widespread recreational and medical use.^4^

Cocaine and cannabis both have well-established cardiovascular effects, but their pharmacologic characteristics and underlying pathophysiological mechanisms are very different. Cocaine has intense sympathomimetic effects and exerts an inhibition of the reuptake of norepinephrine, dopamine, and serotonin, resulting in an increased adrenergic stimulation. This leads to an increase in heart rate, blood pressure, coronary vasoconstriction, increased myocardial oxygen demand, and exaggerated platelet activation, which cause an increased risk of myocardial ischaemia, infarction, malignant arrhythmias, and sudden cardiac death.^5^ There has also been an association of cocaine with acceleration of atherosclerotic disease, loss of endothelial function, and direct myocardial toxicity.^6^

In contrast, the effects of cannabis on the cardiovascular system occur through activation of cannabinoid receptors (CB1 and CB2).^7^ Acute cannabis use has been linked with tachycardia, hypertension or hypotension episodes, elevation of myocardial oxygen demand, as well as abnormalities of coronary microcirculation. There is a new finding of a possible link between cannabis and arrhythmias, myocardial infarction (MI), cerebrovascular accident, and cardiomyopathy among young patients without traditional risk factors. However, the findings of these studies are not as clearly defined as those found in cocaine.

Despite increasing recognition of the cardiovascular consequences of both substances, few studies have explored the comparative effect of one substance vs. another in relation to its effects on cardiovascular problems. The vast majority of studies conducted previously have addressed one substance only, leaving significant gaps in understanding comparative risks. A clearer delineation of these risks is particularly important given the rising prevalence of cannabis use and the persistent burden of cocaine-related cardiovascular events.

Therefore, this study proposes to fill this knowledge gap by assessing and comparing the effect of cocaine use and cannabis use on the provided cardiac outcomes. We will specifically study the incidence of new-onset cardiac arrhythmias, cardiac arrest, major adverse cardiac events such as MI and stroke, and all-cause mortality events due to cocaine use and cannabis use. By directly comparing these substances, this analysis seeks to provide clinically relevant insights that may inform risk stratification, patient counselling, and future public health strategies.

## Methods

### Data selection

This study was conducted using the TriNetX research network, a federated database providing access to electronic health records (EHRs) from 159 healthcare organizations (HCOs). A total of 120 providers responded with patients. The TriNetX platform aggregates de-identified patient data, ensuring compliance with HIPAA de-identification standards (§164.514(a)). The use of anonymized secondary data did not require formal ethical approval, and a waiver of informed consent was granted (or was deemed not required).

### Patient population

We conducted a retrospective observational multicentric cohort study including patients without previous history of cardiac arrhythmia, categorized based on the substance they were using, either cocaine or cannabis. Cohort 1 (cocaine group) included patients without previous history of cardiac arrhythmia who were using cocaine with no history of cannabis use, while Cohort 2 (cannabis group) included patients without previous history of cardiac arrhythmia who were using cannabis with no history of cocaine use. The index event was defined as the diagnosis of cocaine or cannabis use, identified using ICD-10 codes F14.9, F14.2, or F14.10 (cocaine use) and ICD-10 codes F12.1, F12.2, F12.9, or F12.120 (cannabis use), marked the beginning of the observation window for evaluating outcomes. Cardiac arrhythmia diagnosis and cocaine or cannabis use were identified using ICD-10 codes. Additional details on cohort identification and study window definitions, including the relevant ICD-10, RxNorm, and Current Procedural Terminology codes, are provided in the Supplementary Appendix. Data were collected between 17 October 2005 and 17 October 2025. All individuals who met the predefined inclusion criteria during this period were included in the study and were followed from cohort entry until the occurrence of the outcome of interest, end of data availability, or censoring, as appropriate.

### Study objectives

This study aims to compare the cardiovascular outcome between cocaine and cannabis users. The primary outcome of interest was the incidence of new-onset cardiac arrhythmia after initial substance use (cocaine or cannabis). Secondary outcomes included cardiac arrest events, all-cause mortality, and occurrence of major adverse cardiovascular events (MI and stroke). The Supplementary Appendix elaborates on outcome definitions and ICD-10 codes.

### Statistical analysis

Continuous variables are presented as mean ± standard deviation (SD), whereas categorical variables are presented as number (percentage), as appropriate. Baseline characteristics were compared between the cocaine and cannabis groups using independent samples Student’s *t*-tests for continuous variables and *χ*² tests for categorical variables. To mitigate baseline differences between cohorts, 1:1 propensity score matching (PSM) was performed using greedy nearest neighbour matching with a calliper of 0.1 times the pooled SD of the linear propensity scores. Variables included in the matching process were age, sex, race, comorbidities (hypertension, hyperlipidaemia, diabetes mellitus, cardiomyopathy, ischaemic heart disease, cerebrovascular disease, thyroid gland disease, pulmonary disease, obesity, opioid-related disorders, alcohol-related disorders, and nicotine dependence), medication use (aspirin, B-blocker, calcium channel blocker, antilipemic agents, and antiarrhythmic agents), and laboratory values (cholesterol level, haemoglobin A1C, urea, creatinine, troponin I, ferritin, and C-reactive protein). The standardized mean difference represents the difference between the means of two groups in terms of SD units and is used to assess balance in measured variables in the sample weighted by the inverse probability of treatment. Variables were selected based on their potential impact on overall and cardiovascular outcomes.

After PSM, adjusted outcomes were compared between cohorts using hazard ratios (HRs) and 95% confidence intervals (CIs) derived from Cox proportional hazards regression models. Kaplan–Meier survival analysis was used to assess time-to-event outcomes, with differences between cohorts evaluated using the log-rank test. A *P* < 0.05 was considered statistically significant. All statistical analyses were conducted using integrated R (the R Foundation) within the TriNetX platform.

## Results

### Study population

**Figure 1:**
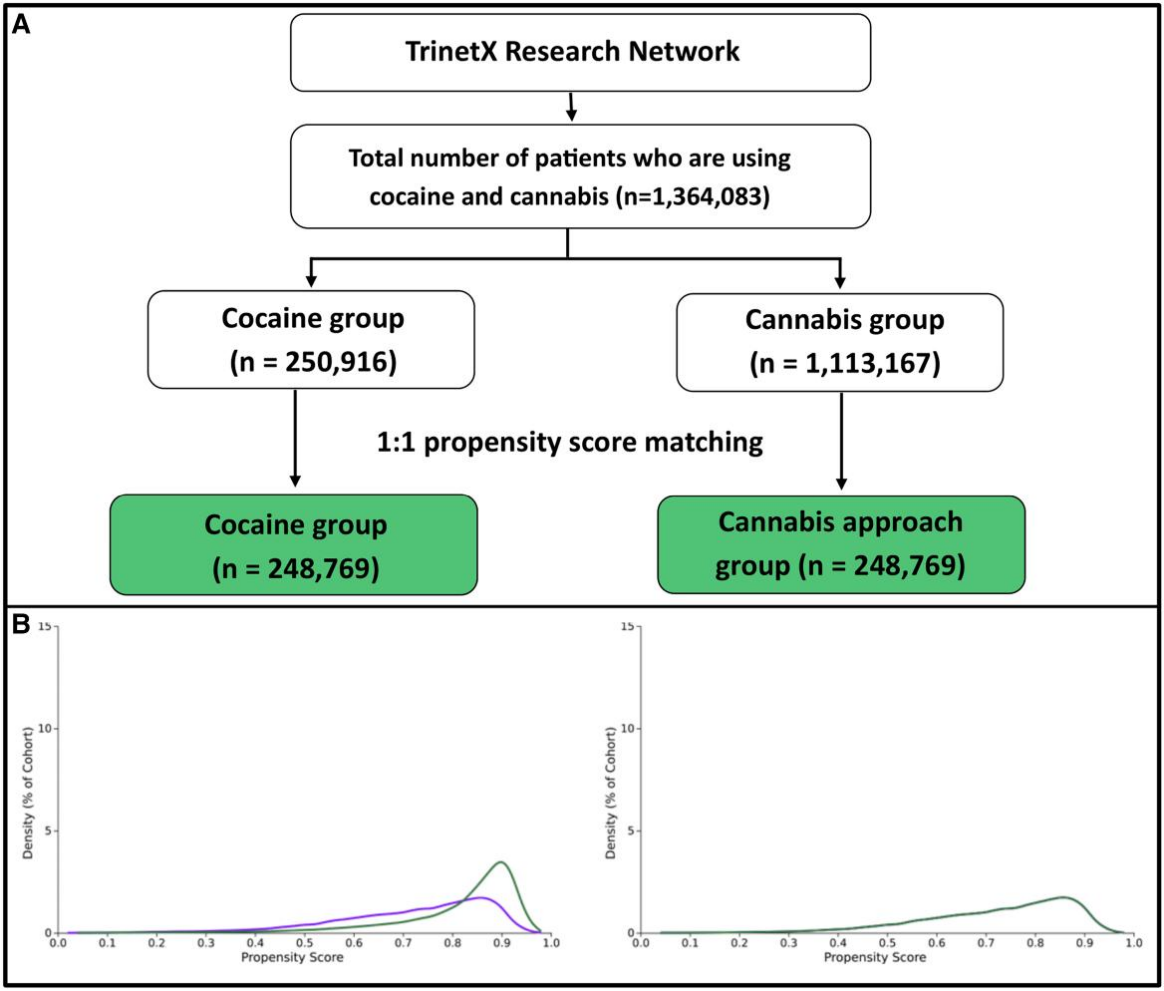
(*A*) Flow diagram of patient selection and cohort derivation following propensity score matching. (*B*) Propensity score density distributions before and after weighting.

This retrospective cohort study identified a total of 1 364 083 patients using cocaine or cannabis and had *no prior history* of cardiac arrhythmia. Among them, 250 916 patients are using cocaine and 1 113 167 patients are using cannabis (*Figure 1A*). After applying 1:1 PSM to balance baseline characteristics, 248 769 patients were included in each cohort (cocaine and cannabis groups) for the final analysis (*Figure 1B*).

### Patient characteristics

The baseline characteristics of the study cohorts, before and after PSM, are shown in *Table 1*. In the unmatched cohort, patients using cocaine were older at index (mean age: 42.6 ± 13.1 years vs. 33.4 ± 15.0 years, *P* < 0.001) compared with those using cannabis. The proportion of male patients was slightly higher in the cocaine group (63.1% vs. 56.4%, *P* < 0.001), while the cannabis group had a higher proportion of female patients (43.5% vs. 36.1%, *P* < 0.001). Regarding racial distribution, the cannabis group was more likely to be White (57.6% vs. 51.1%) compared with the cocaine group.

**Table 1:** Baseline characteristics of patients in cocaine and cannabis groups before and after propensity score matching (PSM)

	Before PSM			After PSM		
	Before matching (cocaine group, *n* = 250 916)	Before matching (cannabis group, *n* = 1 113 167)	Standardized difference	After matching (cocaine group, *n* = 248 769)	After matching (cannabis group, *n* = 248 769)	Standardized difference
Demographics						
Age at index (mean ± SD)	42.6 ± 13.1	33.4 ± 15.0	0.654	42.5 ± 13.0	43.3 ± 14.6	0.061
Female (%)	90 676 (36.1%)	484 619 (43.5%)	0.151	90 157 (36.2%)	90 033 (36.2%)	0.001
Male (%)	160 076 (63.8%)	627 902 (56.4%)	0.151	158 480 (63.7%)	158 513 (63.7%)	0.003
White (%)	128 109 (51.1%)	641 107 (57.6%)	0.131	127 569 (51.3%)	139 392 (56.0%)	0.012
Black or African American (%)	80 575 (32.1%)	286 506 (25.7%)	0.141	79 338 (31.9%)	64 935 (26.1%)	0.128
Comorbid conditions						
Hypertension	47 136 (18.8%)	146 640 (13.2%)	0.154	46 205 (18.6%)	45 493 (18.3%)	0.007
Dyslipidaemia	23 102 (9.2%)	101 354 (9.1%)	0.004	22 901 (9.2%)	23 150 (9.3%)	0.003
Ischaemic heart diseases	10 902 (4.3%)	31 369 (2.8%)	0.082	10 691 (4.3%)	10 457 (4.2%)	0.005
Cardiomyopathy	2380 (0.9%)	6148 (0.6%	0.046	2299 (0.9%)	2224 (0.9%)	0.003
Diabetes mellitus	20 250 (8.1%)	60 115 (5.4%)	0.107	19 772 (7.9%)	19 391 (7.8%)	0.006
Overweight, obesity, and other hyperalimentation	17 567 (7.0%)	110 862 (10.0%)	0.106	17 468 (7.0%)	16 731 (6.7%)	0.012
Diseases of the respiratory system	69 201 (27.6%)	346 925 (31.2%)	0.079	68 311 (27.5%)	66 118 (26.6%)	0.020
Disorders of thyroid gland	8830 (3.5%)	47 444 (4.3%)	0.038	8772 (3.5%)	8995 (3.6%)	0.005
Cerebrovascular diseases	7923 (3.2%)	22 878 (2.1%)	0.069	7769 (3.1%)	7635 (3.1%)	0.003
Nicotine dependence	62 196 (24.8%)	224 627 (20.2%)	0.111	61 102 (24.6%)	61 511 (24.7%)	0.004
Alcohol-related disorders	33 508 (13.4%)	78 978 (7.1%)	0.208	32 505 (13.1%)	32 503 (13.1%)	0.001
Opioid-related disorders	29 848 (11.9%)	34 131 (3.1%)	0.340	27 944 (11.2%)	25 554 (10.3%)	0.031
Medication use						
Aspirin	22 386 (8.9%)	73 894 (6.6%)	0.085	21 990 (8.8%)	21 260 (8.5%)	0.010
Beta-blockers	24 539 (9.8%)	101 990 (9.2%)	0.021	24 245 (9.7%)	24 263 (9.8%)	<0.001
Calcium-channel blockers	18 567 (7.4%)	58 197 (5.2%)	0.089	18 144 (7.3%)	17 545 (7.1%)	0.009
Antilipemic agents	21 177 (8.4%)	78 072 (7.0%)	0.053	20 938 (8.4%)	20 546 (8.3%)	0.006
Antiarrhythmics	49 572 (19.8%)	259 019 (23.3%)	0.086	49 109 (19.7%)	50 272 (20.2%)	0.012
Laboratory						
Troponin I, ng/mL	0.4 ± 4.5	0.4 ± 7.8	0.005	0.4 ± 4.6	0.3 ± 6.8	0.004
Total cholesterol, md/dL	174.5 ± 48.2	173.4 ± 45.9	0.023	174.6 ± 48.2	175.8 ± 47.4	0.024
Haemoglobin A1c, %	6.3 ± 2.1	5.9 ± 1.7	0.228	6.7 ± 2.1	6.1 ± 1.7	0.122
C-reactive protein, mg/dL	33.7 ± 56.4	24.5 ± 48.7	0.176	33.8 ± 56.6	27.2 ± 51.2	0.121
Ferritin, ng/mL	285.2 ± 1359.5	233.3 ± 2126.7	0.029	282.8 ± 1346.7	282.4 ± 1293.0	<0.001
Blood urea nitrogen, mg/dL	13.8 ± 7.9	12.7 ± 6.5	0.155	13.8 ± 7.9	13.5 ± 7.3	0.039
Creatinine, mg/dL	1.2 ± 4.8	1.1 ± 5.2	0.016	1.2 ± 4.7	1.3 ± 6.7	0.026

Before matching, the cocaine patient group exhibited a higher prevalence of cardiovascular risk factors, including cardiomyopathy (0.9% vs. 0.6%, *P* < 0.001), ischaemic heart disease (4.3% vs. 2.8%, *P* < 0.001), cerebrovascular disease (3.2% vs. 2.1%, *P* < 0.001), hypertension (18.8% vs. 13.2%, *P* < 0.001), diabetes mellitus (8.1% vs. 5.4%, *P* < 0.001), nicotine dependence (24.8% vs. 20.2%, *P* < 0.001), alcohol-related disorders (13.4% vs. 7.1%, *P* < 0.001), and opioid-related disorders (11.9% vs. 3.1%, *P* < 0.001). Conversely, overweight and obesity (10.0% vs. 7.0%, *P* < 0.001), pulmonary disease (31.2% vs. 27.6%, *P* < 0.001), and thyroid gland disease (4.3% vs. 3.5%, *P* < 0.001) were more frequently observed in the cannabis patient group. The prevalence of hyperlipidaemia was nearly the same between the two groups (9.2% vs. 9.1%, *P* < 0.001).

After PSM, the two cohorts were well balanced across key baseline characteristics, including age, sex, race, comorbidities, medication use, and laboratory values with standardized mean differences (SMDs) < 0.1 for most variables, indicating a well-matched cohort. The final matched cohort consisted of 250 916 patients in the cocaine group and 250 916 patients in the cannabis group.

### Primary outcome: incident of new-onset cardiac arrhythmia

**Figure 2:**
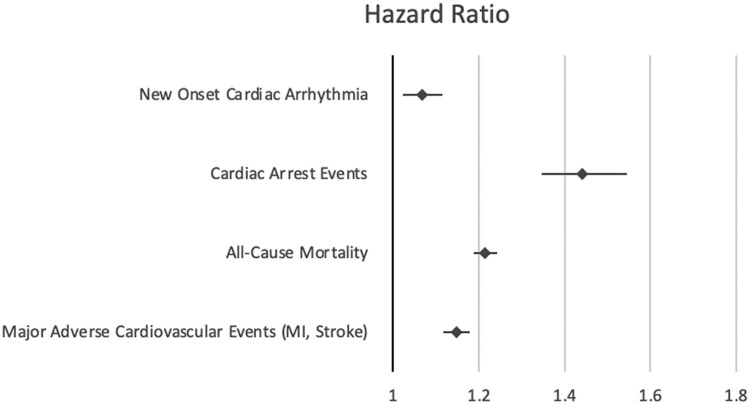
Forest plot of hazard ratios for primary and secondary outcomes.

After matching, during the follow-up period, a total of 4821 patients (0.2%) in the cocaine cohort experienced new-onset cardiac arrhythmia, compared with 3696 patients (0.15%) in the cannabis cohort. Using cocaine was associated with a significantly higher risk of new-onset cardiac arrhythmia with a HR of 1.067 (95% CI: 1.022–1.115, *P* < 0.035). (*Table 2* and *Figure 2*). Even small relative increases in risk can translate into a substantial absolute burden at the population level, particularly given the large number of individuals exposed to cocaine and the serious clinical implications of cardiac arrhythmias. In our study, which included over 500 000 matched patients, this modest increase corresponded to a significantly higher number of arrhythmic events in the cocaine cohort compared with the cannabis cohort. The observed effect size is consistent with prior large-scale observational studies examining arrhythmia risk associated with cocaine use, which have similarly reported modest but statistically significant increases in risk. This consistency across studies supports the biological plausibility of the association.

**Table 2:** Primary and secondary clinical outcomes: cocaine vs. cannabis users

Outcome	Risk of event, % (cocaine group)	Risk of event, % (cannabis group)	Hazard ratio (95% CI)	*P*-value
Primary outcome				
New-onset cardiac arrhythmia	4821 (0.2%)	3696 (0.15%)	1.067 (1.022–1.115)	*P* < 0.001
Secondary outcome				
Cardiac arrest events	2210 (0.9%)	1307 (0.5%)	1.442 (1.346–1.545)	*P* < 0.001
All-cause mortality	17 979 (7.3%)	12 520 (5.0%)	1.215 (1.187–1.243)	*P* < 0.001
Major adverse cardiovascular events (MI, stroke)	12 307 (5.4%)	9216 (4.0%)	1.147 (1.116–1.178)	*P* < 0.001

### Secondary outcome: cardiac arrest events, all-cause mortality, and major adverse cardiovascular events (myocardial infarction and stroke)

After matching, the secondary outcomes demonstrated significant differences between the cocaine group and the cannabis group. Cardiac arrest events were more frequently reported in the cocaine group, with a HR of 1.442 (95% CI: 1.346–1.545, *P* < 0.001). All-cause mortality was also more frequently reported in the cocaine group with a HR of 1.215 (95% CI: 1.187–1.243, *P* < 0.013). Major adverse cardiovascular events were more commonly reported in the cocaine group, with a HR of 1.147 (95% CI: 1.116–1.178, *P* < 0.001) (*Table 2* and *Figure 2*).

## Discussion

Our study found that the risk of developing new-onset cardiac arrhythmia is significantly higher in patients using cocaine compared with patients using cannabis. Although the existing medical literature lacks large prospective studies or clinical trials that compare cocaine and cannabis regarding arrhythmia risk, prior studies support our findings. Recent data showed that the risk of ventricular arrhythmia in cocaine users is higher compared with non-user (adjusted HR 1.15; 95% CI 1.10–1.19).^8^ Another study showed that the risk of ventricular arrhythmia is higher in cannabis users with a HR of 3.08 (95% CI: 2.09–4.53) compared with ibuprofen users.^9^

A population-based study from California showed that the adjusted HR for incidence of atrial fibrillation was 1.61 (95% CI: 1.55–1.68) for cocaine and 1.35 (95% CI: 1.30–1.40) for cannabis, suggesting a higher risk of atrial fibrillation with cocaine compared with cannabis which aligned with our finding.^10^ However, that study investigated the risk of atrial fibrillation without the risk of other arrhythmia, while our study evaluated the broader spectrum of new-onset cardiac arrhythmias.

We also found that the risk of cardiac arrest and major adverse cardiovascular events, including MI and stroke, is significantly higher in cocaine users compared with cannabis users, which is supported by prior literature showing that cocaine use increases the risk of sudden cardiovascular death more than four-fold (OR 4.10; 95% CI 1.12–15.0).^9^ Also, cocaine use has been linked to increase the risk of MI to seven-fold higher compared with non-user,^11^ whereas cannabis users have an estimated risk ratio of 1.29 (95% CI 1.05 to 1.59) for acute coronary syndrome.^12^ Direct comparisons indicate that cocaine confers a greater acute MI risk than cannabis, with odds ratios for MI hospitalization in young adults of 3.9 for cocaine vs. 1.3 for cannabis.^13^

Regarding stroke, recent data showed that the risk is higher with cocaine use as it will increase the risk for haemorrhagic and ischaemic stroke approximately five-fold (OR ≈ 5.05) compared with non-users.^14^ Although cannabis use also has been linked to increased risk of stroke, the magnitude is lower than that for cocaine, as frequent cannabis use (e.g. >10 days/month) in young adults is associated with an adjusted odds ratio of 2.45 for stroke compared with non-users.^15^ For all-cause mortality, our study found that it is more frequent in cocaine users compared with cannabis users, which is consistent with data that we have in the current literature as direct comparison confirms that cocaine users have a higher all-cause mortality risk than cannabis users.^16^

Cocaine has arrhythmogenic effects through several mechanisms, including blockade of sodium and calcium channel in cardiac myocytes and increased sympathetic flow to the heart by decreased catecholamine reuptake; also cocaine induces vasospasm in the coronary artery which induced cardiac ischaemia.^17,18^ Cannabis also induces arrhythmia through autonomic dysregulation by decreased parasympathetic flow and increased sympathetic flow.^19^ Moreover, cannabis can induce a channelopathy effect on cardiac myocytes by modulating potassium channels by inhibition.^20^ The previously mentioned mechanism could explain elevated risk of arrhythmia with both substances.

### Limitations of the study

Our study has limitations like other observational retrospective studies. It cannot establish causality. Despite PSM helped reduce confounding, unmeasured factors such as quantity, frequency, and type of cocaine and cannabis use, physical activity, healthcare accessibility, and socioeconomic status may still have influenced the results. Additionally, our data analysis did not include information about dose or mode of cocaine or cannabis use. Therefore, our findings cannot establish a dose–response relationship. Also, in our study, substance use status was defined based on the index diagnosis of cocaine or cannabis use identified by ICD-10 codes. Due to the inherent limitations of the TriNetX database, granular longitudinal data regarding persistence, discontinuation, frequency, quantity, or changes in substance use after the index event were not consistently available. As such, we were unable to reliably ascertain whether patients continued, reduced, or discontinued cocaine or cannabis use during follow-up. Furthermore, cocaine or cannabis use disorders were diagnosed based on International Classification of Diseases (ICD) codes, which carries the risk of misclassification or coding errors and biases common in real-world databases.

In regard to these limitations, our study has notable strengths. It is considered one of the first studies to compare cocaine users to cannabis users using a large, real-world dataset spanning diverse healthcare settings, thereby investigating the relationship between use and cardiac arrhythmias across a diverse, multicentre patient population. We employed rigorous PSM to enhance comparability between the cocaine and cannabis cohorts across a wide range of demographic and clinical variables. Although we lacked detailed data on the frequency, quantity, or route of cocaine or cannabis use, our methodology allowed exploration of potential associations between cocaine or cannabis exposure and arrhythmia risk within a large, diverse patient population.

## Conclusion

Our retrospective cohort analysis demonstrates that cocaine users experience significantly higher rates of new-onset cardiac arrhythmias, cardiac arrest, and major adverse cardiovascular events (MI and stroke) compared with cannabis users. These findings highlight the differing outcomes associated with substance use. Future research should aim to validate our findings through prospective, multicentre studies with standardized diagnostic methods as well as longer-term follow-up to more accurately define the outcomes. Our study highlights the importance of recognizing that cocaine and cannabis users carry a significantly higher risk of adverse cardiac outcomes. These findings emphasize the need for early identification, proactive monitoring, and timely intervention to optimize patient care.

## Supplementary Material

zuag011_Supplementary_Data

## Data Availability

The data supporting the findings of this study are available through the TriNetX research network but are subject to licensing restrictions. Access to TriNetX data can be obtained upon reasonable request and with permission from TriNetX, LLC.

## Code availability

Not applicable. No custom code or software was developed or used for this study.
