# Supplementary material for: Incidence of new-onset cardiac arrhythmias in cocaine and cannabis users: a retrospective cohort study
Source: Eur Heart J Acute Cardiovasc Care. 2026 Feb 5;15(6):458–65. doi: 10.1093/ehjacc/zuag011 (PMC13317127; doi:10.1093/ehjacc/zuag011)
Supplement: zuag011_Supplementary_Data [file zuag011_supplementary_data.pdf]

Outcome Definitions

Table below outlines the definitions for each outcome and the analysis specifications. For outcome definitions consisting of more than one term, at least one term must match. Please see Appendix C for the text representation of the outcome definitions.

**Cardiac Arrhythmia**			
**Outcome definition**			
	Diagnosis	UMLS:ICD10CM:I47.29	Other ventricular tachycardia
	Diagnosis	UMLS:ICD10CM:I47.20	Ventricular tachycardia, unspecified
	Diagnosis	UMLS:ICD10CM:I49.0	Ventricular fibrillation and flutter
	Diagnosis	UMLS:ICD10CM:I49.01	Ventricular fibrillation
	Diagnosis	UMLS:ICD10CM:I49.02	Ventricular flutter
	Laboratory	UMLS:LNC:8616-5	Premature ventricular contractions
	Laboratory	UMLS:LNC:8615-7	Premature atrial contractions
	Diagnosis	UMLS:ICD10CM:I49.3	Ventricular premature depolarization
	Diagnosis	UMLS:ICD10CM:I49.1	Atrial premature depolarization
	Diagnosis	UMLS:ICD10CM:I48.0	Paroxysmal atrial fibrillation
	Diagnosis	UMLS:ICD10CM:I47	Paroxysmal tachycardia
	Diagnosis	UMLS:ICD10CM:I47.9	Paroxysmal tachycardia, unspecified
	Diagnosis	UMLS:ICD10CM:I47.1	Supraventricular tachycardia
	Diagnosis	UMLS:ICD10CM:I47.1	Supraventricular tachycardia
	Diagnosis	UMLS:ICD10CM:I47.10	Supraventricular tachycardia, unspecified
	Diagnosis	UMLS:ICD10CM:I49.3	Ventricular premature depolarization
	Diagnosis	UMLS:ICD10CM:I47.19	Other supraventricular tachycardia
	Diagnosis	UMLS:ICD10CM:I48.91	Unspecified atrial fibrillation
	Diagnosis	UMLS:ICD10CM:I48.0	Paroxysmal atrial fibrillation
	Diagnosis	UMLS:ICD10CM:I48.1	Persistent atrial fibrillation
	Diagnosis	UMLS:ICD10CM:I48.2	Chronic atrial fibrillation
	Diagnosis	UMLS:ICD10CM:I48.19	Other persistent atrial fibrillation
	Diagnosis	UMLS:ICD10CM:I48.21	Permanent atrial fibrillation
	Diagnosis	UMLS:ICD10CM:I48	Atrial fibrillation and flutter
	Diagnosis	UMLS:ICD10CM:I48.20	Chronic atrial fibrillation, unspecified
	Diagnosis	UMLS:ICD10CM:I48.11	Longstanding persistent atrial fibrillation
	Diagnosis	UMLS:ICD10CM:I48.92	Unspecified atrial flutter
	Diagnosis	UMLS:ICD10CM:I48.3	Typical atrial flutter
	Diagnosis	UMLS:ICD10CM:I48.4	Atypical atrial flutter
	Diagnosis	UMLS:ICD10CM:Z86.79	Personal history of other diseases of the circulatory system
	Diagnosis	UMLS:ICD10CM:I48.9	Unspecified atrial fibrillation and atrial flutter
**Settings for the performed analyses**			
	Risk analysis		excluding patients with outcome prior to the time window
	Kaplan - Meier survival analysis		excluding patients with outcome prior to the time window
**cardiac arrest**			
**Outcome definition**			
	Diagnosis	UMLS:ICD10CM:I46	Cardiac arrest
**Settings for the performed analyses**			
	Risk analysis		excluding patients with outcome prior to the time window
	Kaplan - Meier survival analysis		excluding patients with outcome prior to the time window
**All cause mortality**			
**Outcome definition**			
	Demographics	Deceased	Deceased
**Settings for the performed analyses**			
	Risk analysis		excluding patients with outcome prior to the time window
	Kaplan - Meier survival analysis		excluding patients with outcome prior to the time window
**Major adverse cardiovascular events**			
**Outcome definition**			

Diagnosis	UMLS:ICD10CM:I21.4	Non-ST elevation (NSTEMI) myocardial infarction
Diagnosis	UMLS:ICD10CM:I22.2	Subsequent non-ST elevation (NSTEMI) myocardial infarction
Diagnosis	UMLS:ICD10CM:I20.0	Unstable angina
Diagnosis	UMLS:ICD10CM:I24.8	Other forms of acute ischemic heart disease
Diagnosis	UMLS:ICD10CM:I21.3	ST elevation (STEMI) myocardial infarction of unspecified site
Diagnosis	UMLS:ICD10CM:I21.29	ST elevation (STEMI) myocardial infarction involving other sites
Diagnosis	UMLS:ICD10CM:I21.11	ST elevation (STEMI) myocardial infarction involving right coronary artery
Diagnosis	UMLS:ICD10CM:I21.09	ST elevation (STEMI) myocardial infarction involving other coronary artery of anterior wall
Diagnosis	UMLS:ICD10CM:I21.21	ST elevation (STEMI) myocardial infarction involving left circumflex coronary artery
Diagnosis	UMLS:ICD10CM:I21.01	ST elevation (STEMI) myocardial infarction involving left main coronary artery
Diagnosis	UMLS:ICD10CM:I21.1	ST elevation (STEMI) myocardial infarction of inferior wall
Diagnosis	UMLS:ICD10CM:I21.0	ST elevation (STEMI) myocardial infarction of anterior wall
Diagnosis	UMLS:ICD10CM:I21.19	ST elevation (STEMI) myocardial infarction involving other coronary artery of inferior wall
Diagnosis	UMLS:ICD10CM:I21.2	ST elevation (STEMI) myocardial infarction of other sites
Diagnosis	UMLS:ICD10CM:I21.02	ST elevation (STEMI) myocardial infarction involving left anterior descending coronary artery
Diagnosis	UMLS:ICD10CM:I22	Subsequent ST elevation (STEMI) and non-ST elevation (NSTEMI) myocardial infarction
Diagnosis	UMLS:ICD10CM:I21	Acute myocardial infarction
Diagnosis	UMLS:ICD10CM:I60-I69	Cerebrovascular diseases
Diagnosis	UMLS:ICD10CM:I67.9	Cerebrovascular disease, unspecified
Diagnosis	UMLS:ICD10CM:I63	Cerebral infarction
Diagnosis	UMLS:ICD10CM:I63.50	Cerebral infarction due to unspecified occlusion or stenosis of unspecified cerebral artery
**Settings for the performed analyses**		
Risk analysis		excluding patients with outcome prior to the time window
Kaplan - Meier survival analysis		excluding patients with outcome prior to the time window

Appendix A – Text Representation of the Cohorts Definition

This section lists all terms used in the definitions of the two cohorts.

Query Criteria for Cohort 1 (Cocaine Group)

Patients cannot have:
any of the following:
Cardiac arrhythmia, unspecified (UMLS:ICD10CM:I49.9); or
Unspecified atrial fibrillation (UMLS:ICD10CM:I48.91); or
Unspecified atrial flutter (UMLS:ICD10CM:I48.92); or
Supraventricular tachycardia (UMLS:ICD10CM:I47.1); or
Ventricular tachycardia (UMLS:ICD10CM:I47.2); or
Atrial premature depolarization (UMLS:ICD10CM:I49.1); or
Ventricular premature depolarization (UMLS:ICD10CM:I49.3); or
Ventricular fibrillation (UMLS:ICD10CM:I49.01); or
Ventricular fibrillation and flutter (UMLS:ICD10CM:I49.0); or
Paroxysmal tachycardia (UMLS:ICD10CM:I47).

All the following must be satisfied:

Group 1 (Cocaine use): The terms in this group occurred between Oct 17, 2005 and Oct 17, 2025
- Patients must have:
  - any of the following:
    - Cocaine use, unspecified (UMLS:ICD10CM:F14.9); or
    - Cocaine dependence (UMLS:ICD10CM:F14.2); or
    - Cocaine abuse, uncomplicated (UMLS:ICD10CM:F14.10).
- Patients cannot have:
  - any of the following:
    - Cannabis abuse (UMLS:ICD10CM:F12.1); or
    - Cannabis dependence (UMLS:ICD10CM:F12.2); or
    - Cannabis use, unspecified (UMLS:ICD10CM:F12.9); or
    - Cannabis abuse with intoxication, uncomplicated (UMLS:ICD10CM:F12.120).

Query Criteria for Cohort 2 (Cannabis Group)

Patients cannot have:
- any of the following:
  - Cardiac arrhythmia, unspecified (UMLS:ICD10CM:I49.9); or
  - Unspecified atrial fibrillation (UMLS:ICD10CM:I48.91); or
  - Unspecified atrial flutter (UMLS:ICD10CM:I48.92); or
  - Supraventricular tachycardia (UMLS:ICD10CM:I47.1); or
  - Ventricular tachycardia (UMLS:ICD10CM:I47.2); or
  - Ventricular fibrillation (UMLS:ICD10CM:I49.01); or
  - Ventricular fibrillation and flutter (UMLS:ICD10CM:I49.0); or
  - Atrial premature depolarization (UMLS:ICD10CM:I49.1); or
  - Ventricular premature depolarization (UMLS:ICD10CM:I49.3); or
  - Paroxysmal tachycardia (UMLS:ICD10CM:I47).

All the following must be satisfied:

Group (Cannabis use): The terms in this group occurred between Oct 17, 2005 and Oct 17, 2025
- Patients must have:
  - any of the following:
    - Cannabis abuse (UMLS:ICD10CM:F12.1); or
    - Cannabis dependence (UMLS:ICD10CM:F12.2); or
    - Cannabis use, unspecified (UMLS:ICD10CM:F12.9); or
    - Cannabis abuse with intoxication, uncomplicated (UMLS:ICD10CM:F12.120).
- Patients cannot have:
  - any of the following:
    - Cocaine use, unspecified (UMLS:ICD10CM:F14.9); or
    - Cocaine abuse, uncomplicated (UMLS:ICD10CM:F14.10); or
    - Cocaine dependence (UMLS:ICD10CM:F14.2).

Appendix B – Text Representation of the Analysis Setup

This section contains the Index Event definition for each cohort.

The index event for Cohort 1 (Cocaine group) is defined as the following:

All the following must be satisfied:

Group 1 (Cocaine use): The terms in this group occurred between Oct 17, 2005 and Oct 17, 2025
- Patients must have:
  - any of the following:
    - Cocaine use, unspecified (UMLS:ICD10CM:F14.9); or
    - Cocaine dependence (UMLS:ICD10CM:F14.2); or
    - Cocaine abuse, uncomplicated (UMLS:ICD10CM:F14.10).
- Patients cannot have:
  - any of the following:
    - Cannabis abuse (UMLS:ICD10CM:F12.1); or
    - Cannabis dependence (UMLS:ICD10CM:F12.2); or
    - Cannabis use, unspecified (UMLS:ICD10CM:F12.9); or
    - Cannabis abuse with intoxication, uncomplicated (UMLS:ICD10CM:F12.120).

The index event for Cohort 2 (Cannabis) is defined as the following:

All the following must be satisfied:

Group 1 (Cannabis): The terms in this group occurred between Oct 17, 2005 and Oct 17, 2025
- Patients must have:
  - any of the following:
    - Cannabis abuse (UMLS:ICD10CM:F12.1); or
    - Cannabis dependence (UMLS:ICD10CM:F12.2); or
    - Cannabis use, unspecified (UMLS:ICD10CM:F12.9); or
    - Cannabis abuse with intoxication, uncomplicated (UMLS:ICD10CM:F12.120).
- Patients cannot have:
  - any of the following:
    - Cocaine use, unspecified (UMLS:ICD10CM:F14.9); or
    - Cocaine abuse, uncomplicated (UMLS:ICD10CM:F14.10); or
    - Cocaine dependence (UMLS:ICD10CM:F14.2).

Appendix C – Text Representation of the Outcomes Definition

This analysis includes the following outcomes:

Cardiac Arrhythmia
- Patients must have:
  - any of the following:
    - Other ventricular tachycardia (UMLS:ICD10CM:I47.29); or
    - Ventricular tachycardia, unspecified (UMLS:ICD10CM:I47.20); or
    - Ventricular fibrillation and flutter (UMLS:ICD10CM:I49.0); or
    - Ventricular fibrillation (UMLS:ICD10CM:I49.01); or
    - Ventricular flutter (UMLS:ICD10CM:I49.02); or
    - Premature ventricular contractions (UMLS:LNC:8616-5); or
    - Premature atrial contractions (UMLS:LNC:8615-7); or

Ventricular premature depolarization (UMLS:ICD10CM:I49.3); or
Atrial premature depolarization (UMLS:ICD10CM:I49.1); or
Paroxysmal atrial fibrillation (UMLS:ICD10CM:I48.0); or
Paroxysmal tachycardia (UMLS:ICD10CM:I47); or
Paroxysmal tachycardia, unspecified (UMLS:ICD10CM:I47.9); or
Supraventricular tachycardia (UMLS:ICD10CM:I47.1); or
Supraventricular tachycardia (UMLS:ICD10CM:I47.1); or
Supraventricular tachycardia, unspecified (UMLS:ICD10CM:I47.10); or
Ventricular premature depolarization (UMLS:ICD10CM:I49.3); or
Other supraventricular tachycardia (UMLS:ICD10CM:I47.19); or
Unspecified atrial fibrillation (UMLS:ICD10CM:I48.91); or
Paroxysmal atrial fibrillation (UMLS:ICD10CM:I48.0); or
Persistent atrial fibrillation (UMLS:ICD10CM:I48.1); or
Chronic atrial fibrillation (UMLS:ICD10CM:I48.2); or
Other persistent atrial fibrillation (UMLS:ICD10CM:I48.19); or
Permanent atrial fibrillation (UMLS:ICD10CM:I48.21); or
Atrial fibrillation and flutter (UMLS:ICD10CM:I48); or
Chronic atrial fibrillation, unspecified (UMLS:ICD10CM:I48.20); or
Longstanding persistent atrial fibrillation (UMLS:ICD10CM:I48.11); or
Unspecified atrial flutter (UMLS:ICD10CM:I48.92); or
Typical atrial flutter (UMLS:ICD10CM:I48.3); or
Atypical atrial flutter (UMLS:ICD10CM:I48.4); or
Personal history of other diseases of the circulatory system (UMLS:ICD10CM:Z86.79); or
Unspecified atrial fibrillation and atrial flutter (UMLS:ICD10CM:I48.9).

cardiac arrest
Patients must have:
Cardiac arrest (UMLS:ICD10CM:I46).

All cause mortality
Patients must have:
Deceased (Deceased).

Major adverse cardiovascular events
Patients must have:
any of the following:
Non-ST elevation (NSTEMI) myocardial infarction (UMLS:ICD10CM:I21.4); or
Subsequent non-ST elevation (NSTEMI) myocardial infarction (UMLS:ICD10CM:I22.2); or
Unstable angina (UMLS:ICD10CM:I20.0); or
Other forms of acute ischemic heart disease (UMLS:ICD10CM:I24.8); or
ST elevation (STEMI) myocardial infarction of unspecified site (UMLS:ICD10CM:I21.3); or
ST elevation (STEMI) myocardial infarction involving other sites (UMLS:ICD10CM:I21.29); or
ST elevation (STEMI) myocardial infarction involving right coronary artery (UMLS:ICD10CM:I21.11); or
ST elevation (STEMI) myocardial infarction involving other coronary artery of anterior wall (UMLS:ICD10CM:I21.09); or
ST elevation (STEMI) myocardial infarction involving left circumflex coronary artery (UMLS:ICD10CM:I21.21); or
ST elevation (STEMI) myocardial infarction involving left main coronary artery (UMLS:ICD10CM:I21.01); or
ST elevation (STEMI) myocardial infarction of inferior wall (UMLS:ICD10CM:I21.1); or

ST elevation (STEMI) myocardial infarction of anterior wall (UMLS:ICD10CM:I21.0); or

ST elevation (STEMI) myocardial infarction involving other coronary artery of inferior wall (UMLS:ICD10CM:I21.19); or

ST elevation (STEMI) myocardial infarction of other sites (UMLS:ICD10CM:I21.2); or

ST elevation (STEMI) myocardial infarction involving left anterior descending coronary artery (UMLS:ICD10CM:I21.02); or

Subsequent ST elevation (STEMI) and non-ST elevation (NSTEMI) myocardial infarction (UMLS:ICD10CM:I22); or

Acute myocardial infarction (UMLS:ICD10CM:I21); or

Cerebrovascular diseases (UMLS:ICD10CM:I60-I69); or

Cerebrovascular disease, unspecified (UMLS:ICD10CM:I67.9); or

Cerebral infarction (UMLS:ICD10CM:I63); or

Cerebral infarction due to unspecified occlusion or stenosis of unspecified cerebral artery (UMLS:ICD10CM:I63.50).
